# Supplementary figures and images for: Identification of Pathway-Based Biomarkers with Crosstalk Analysis for Overall Survival Risk Prediction in Breast Cancer
Source: Front Genet. 2021 Oct 21;12:689715. doi: 10.3389/fgene.2021.689715 (PMC8566719; doi:10.3389/fgene.2021.689715)

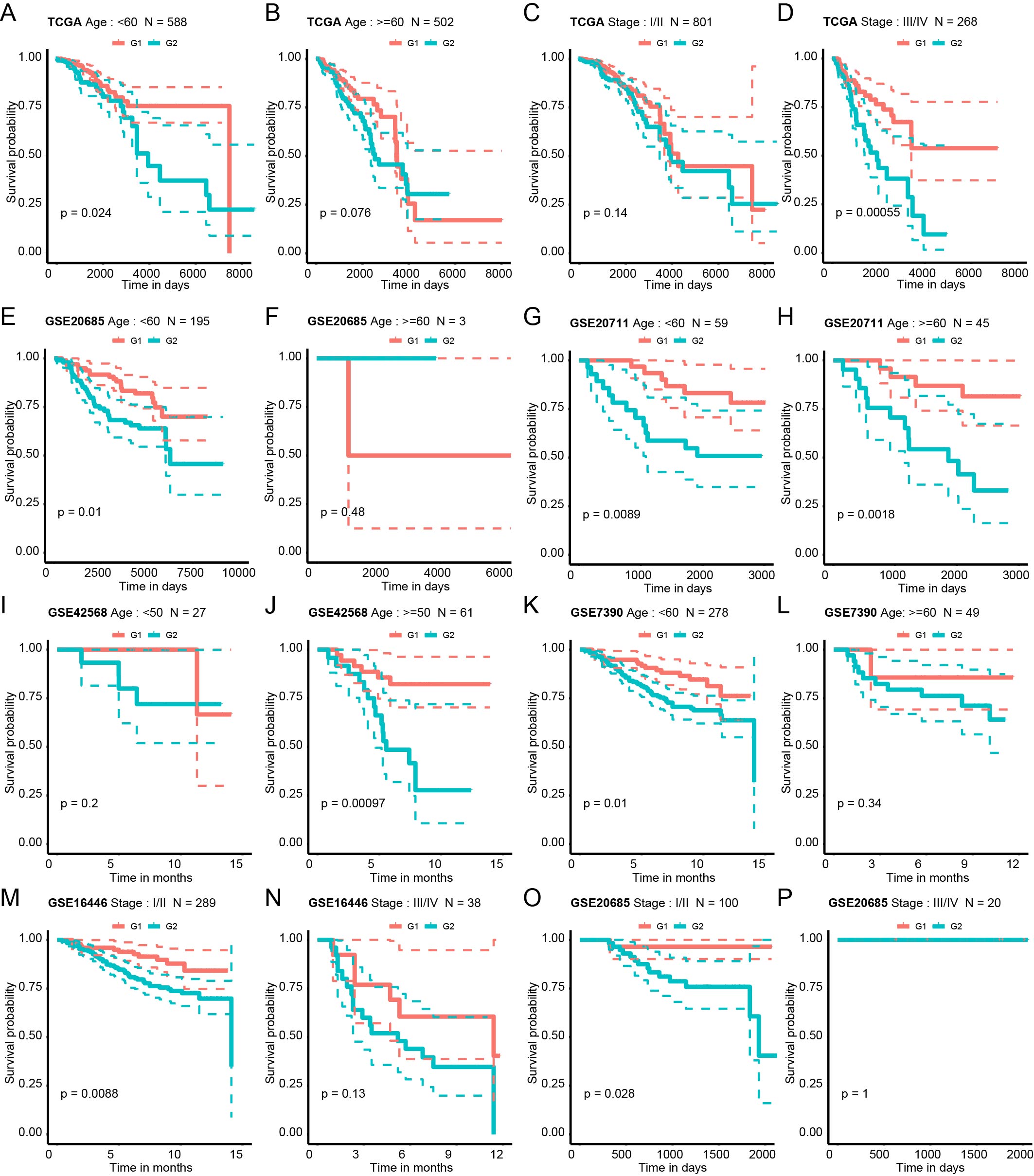

Supplement: Supplementary file 2 [file Image3.JPEG]

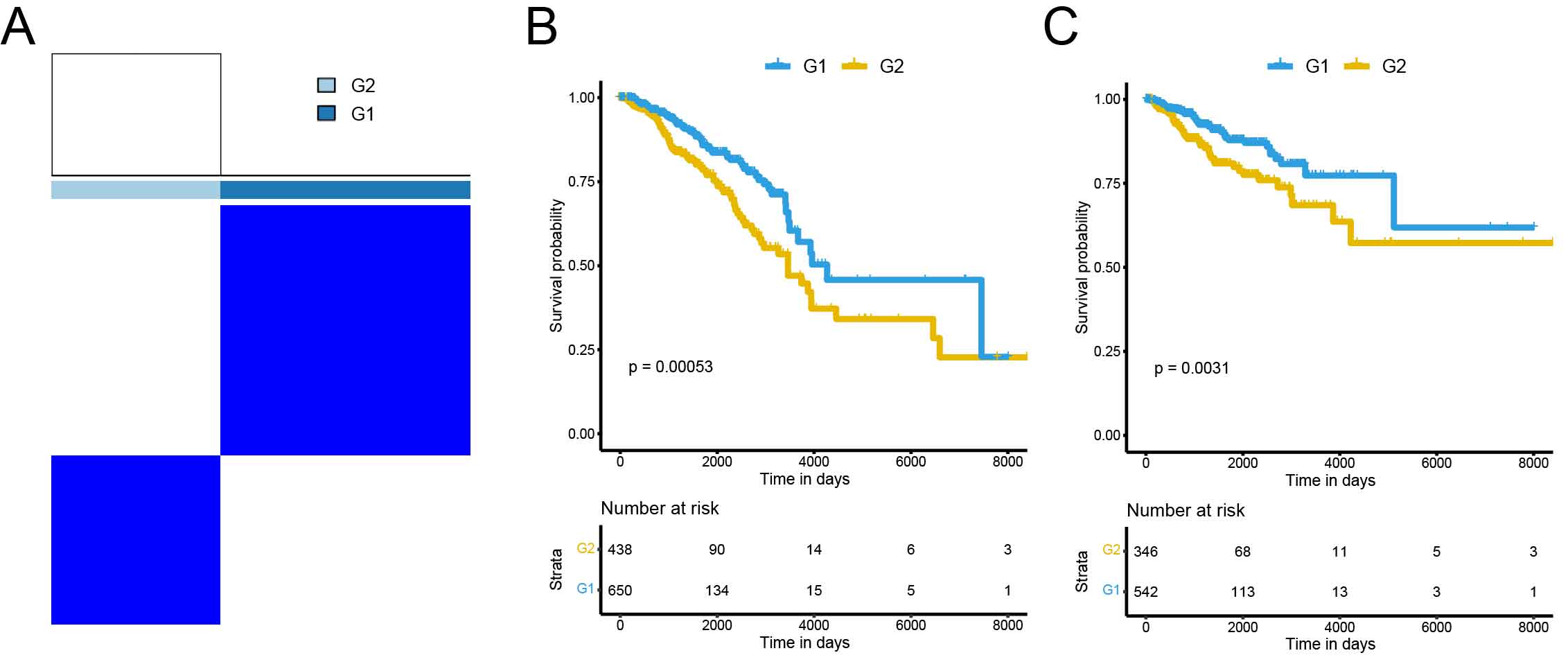

Supplement: Supplementary file 3 [file Image1.JPEG]

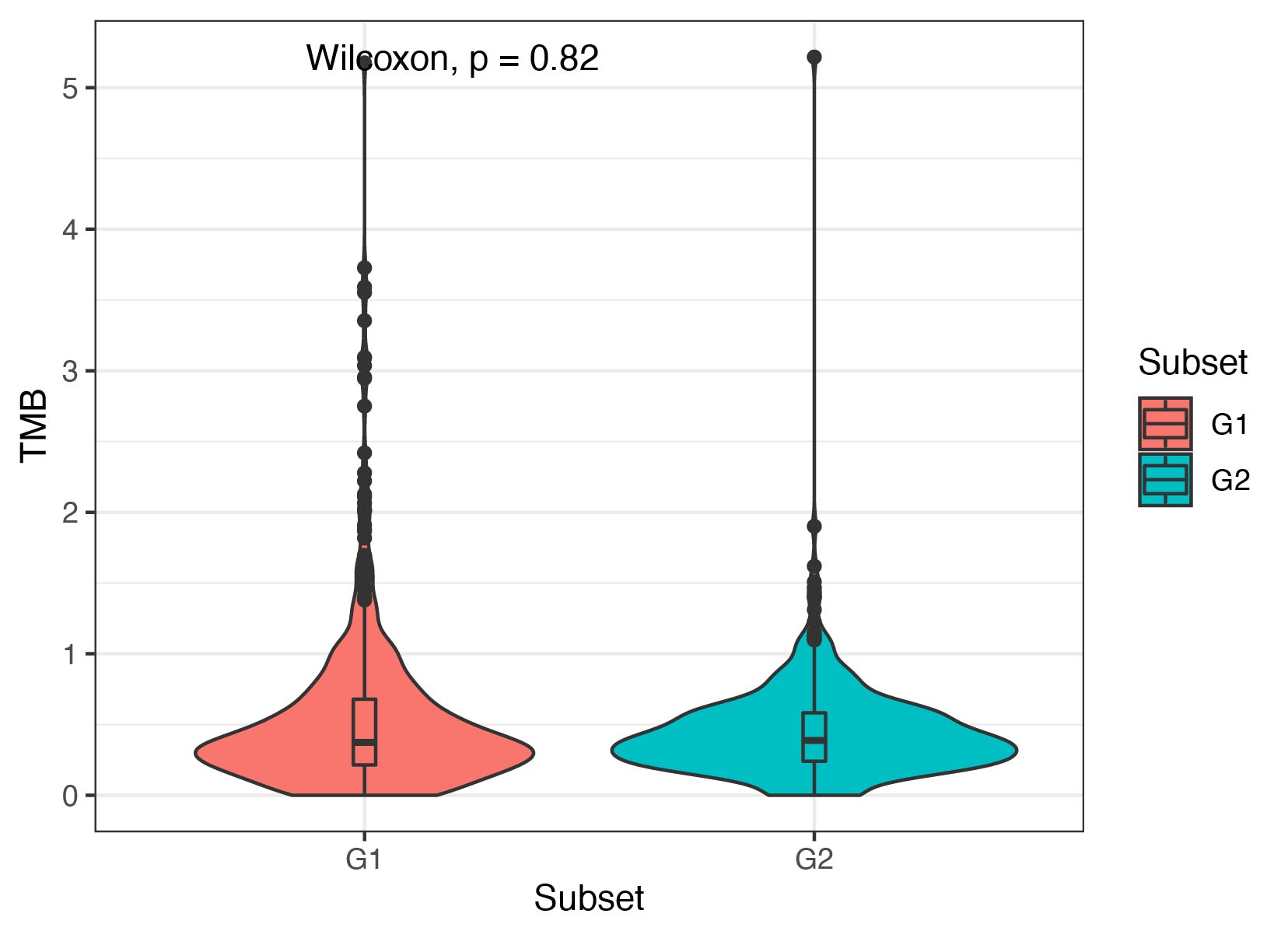

Supplement: Supplementary file 4 [file Image4.JPEG]

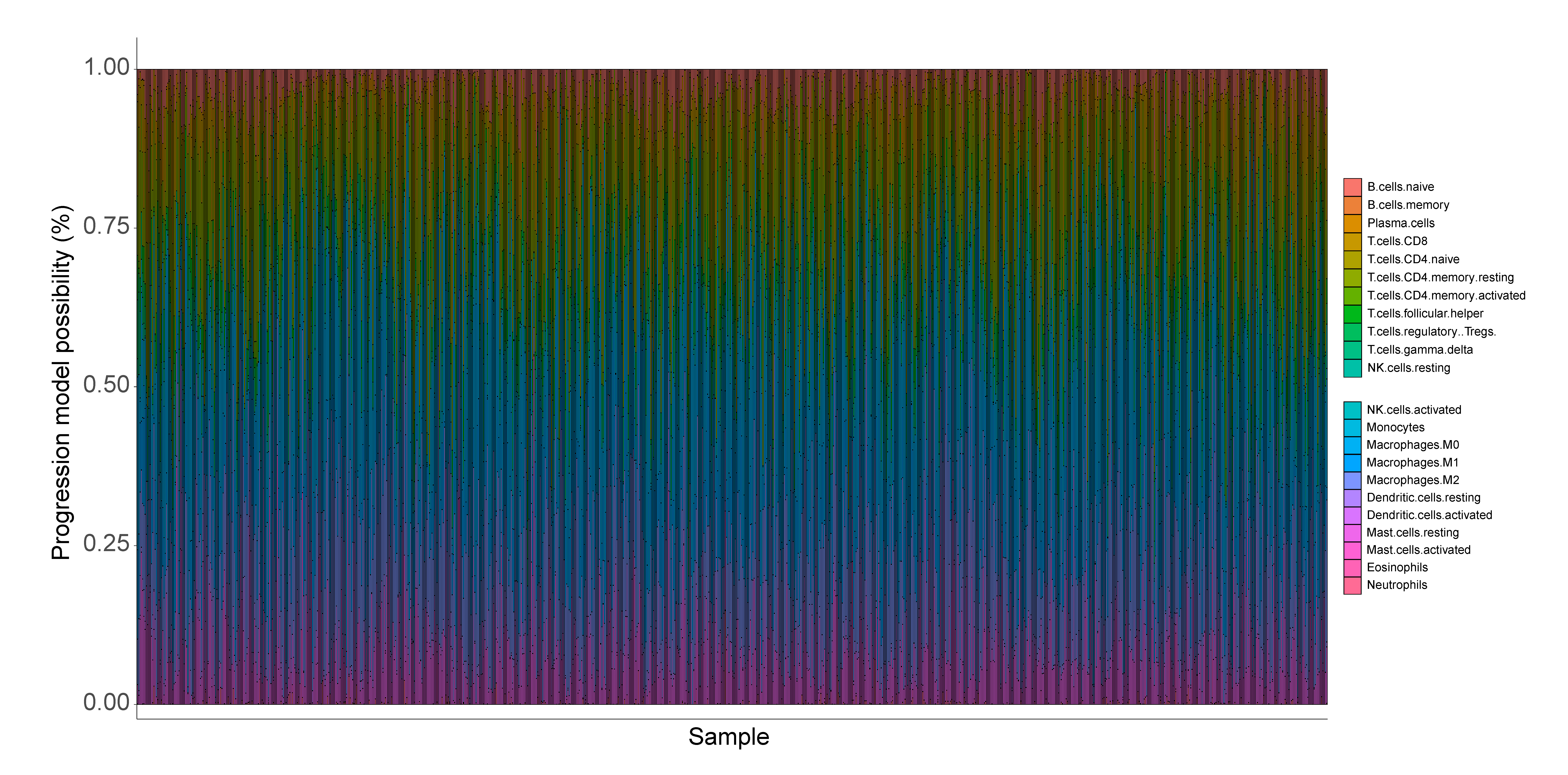

Supplement: Supplementary file 6 [file Image7.JPEG]

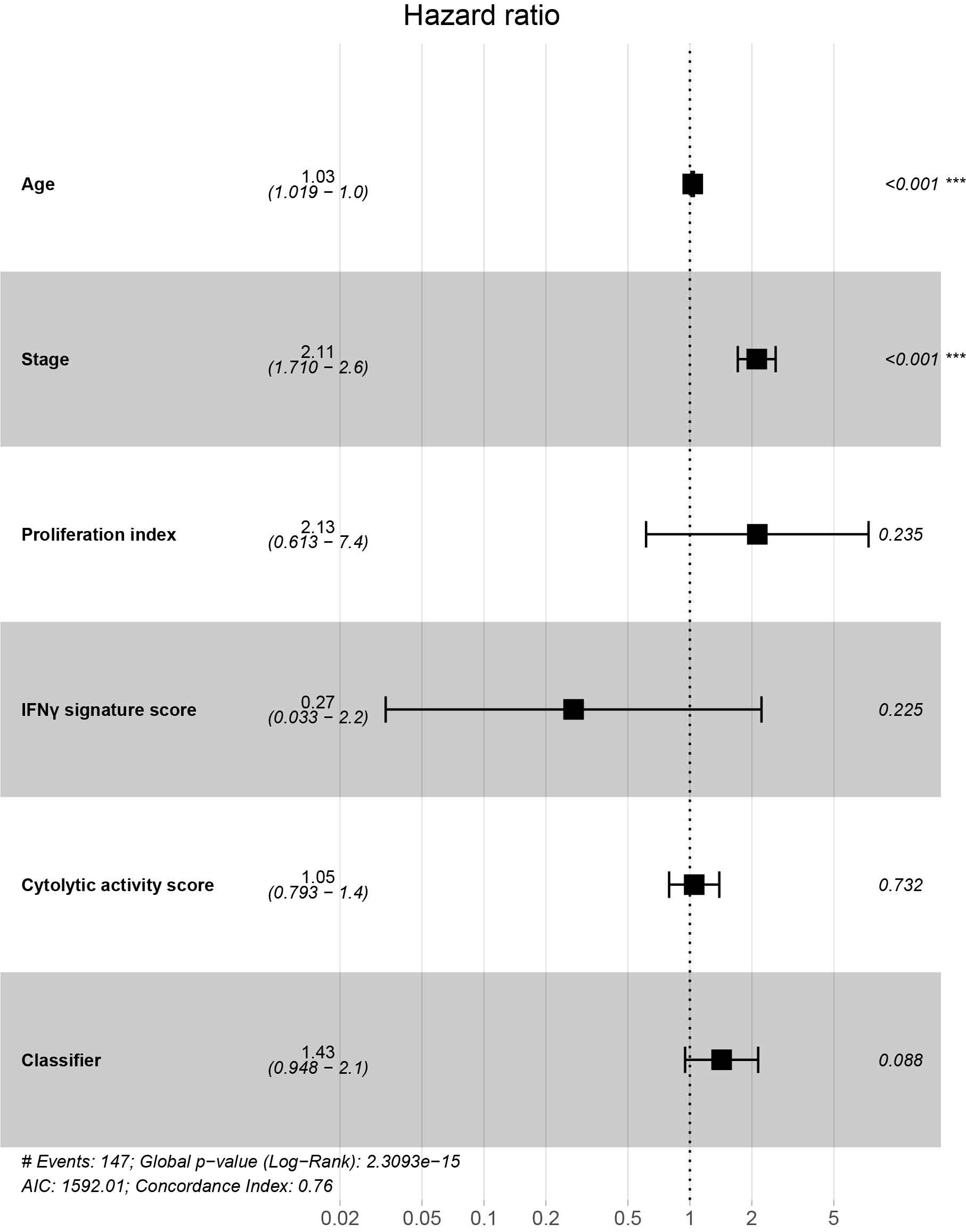

Supplement: Supplementary file 7 [file Image2.JPEG]

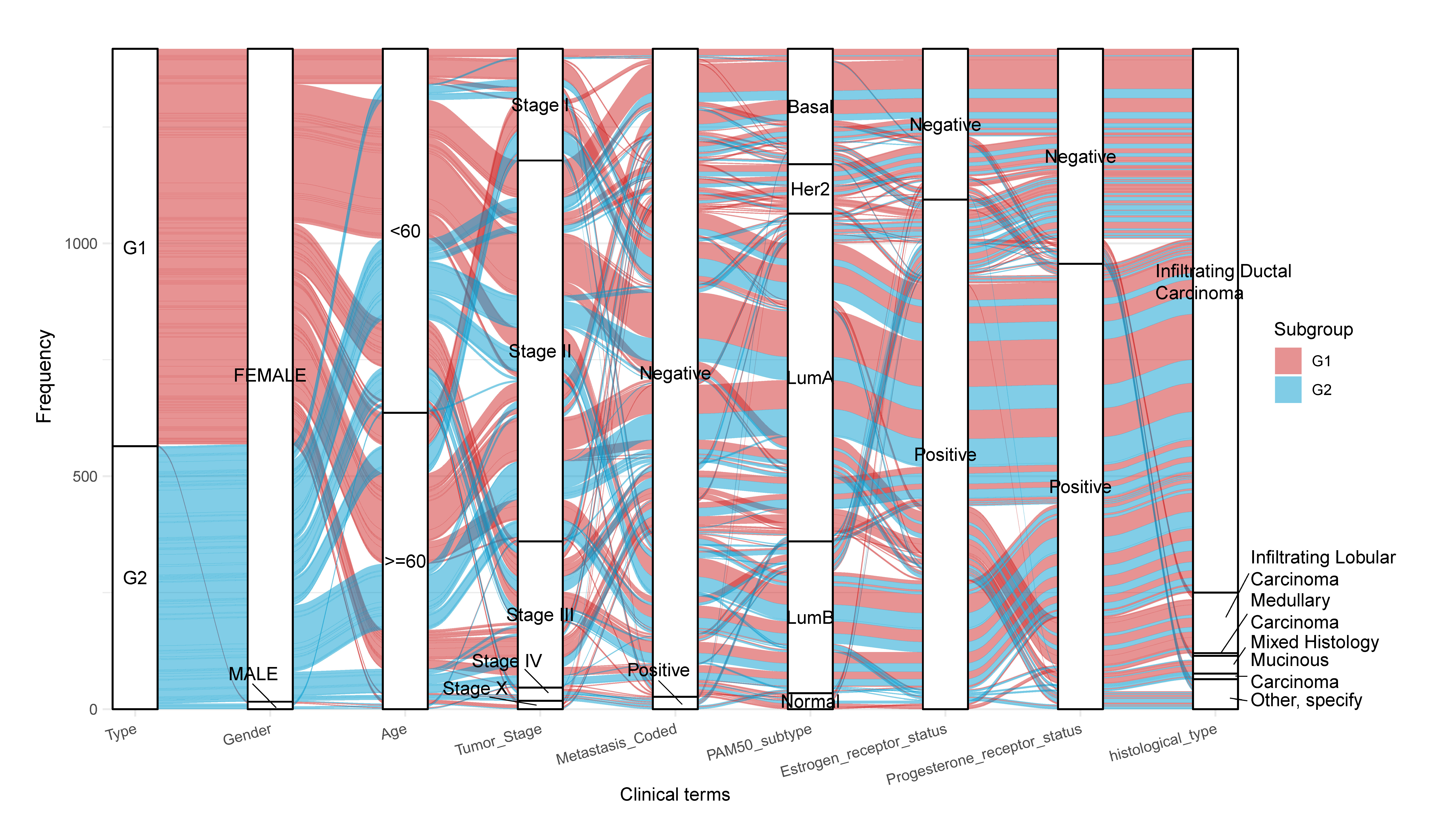

Supplement: Supplementary file 8 [file Image5.JPEG]

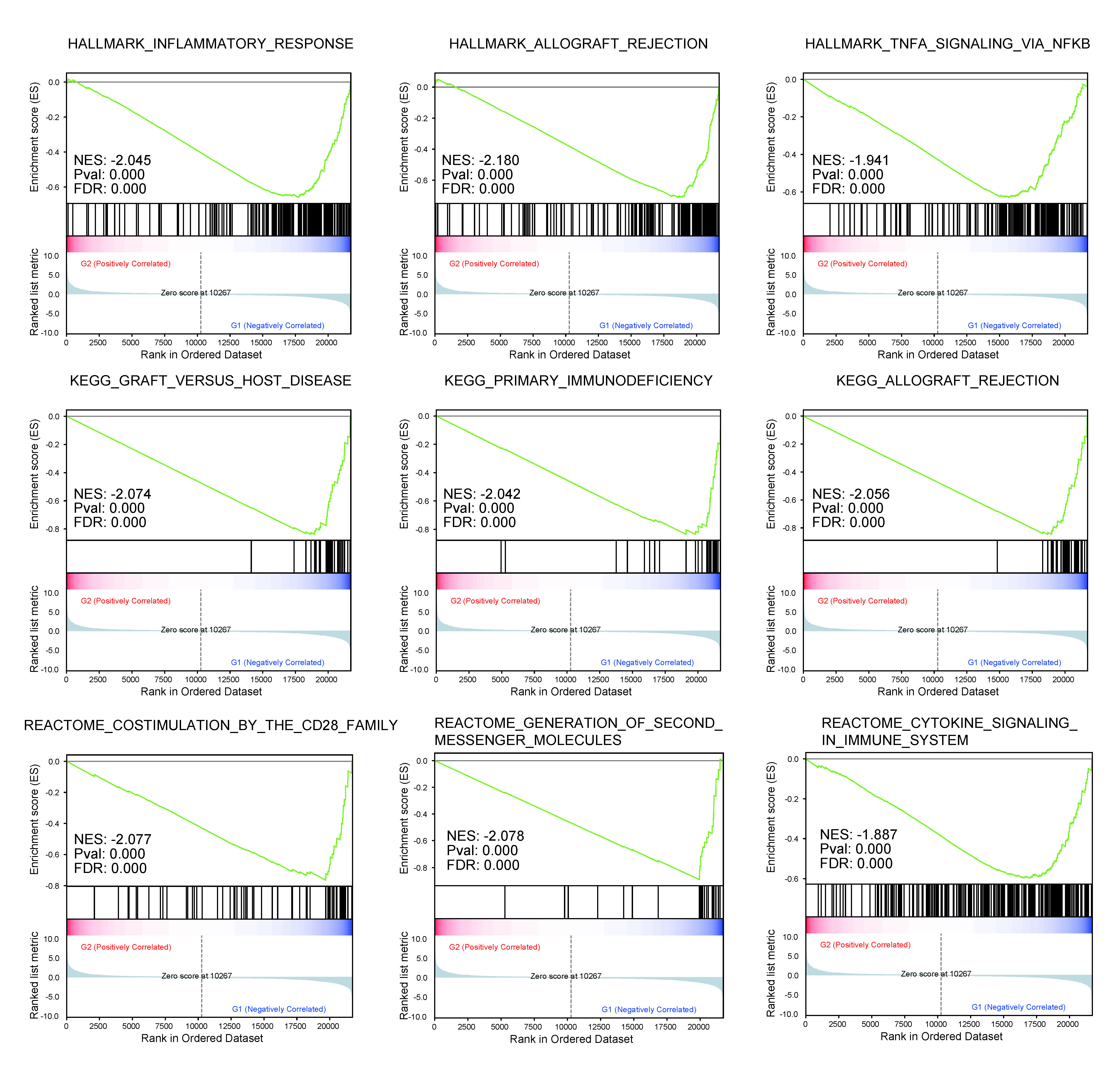

Supplement: Supplementary file 16 [file Image6.JPEG]
