# Supplementary material for: Identification of Pathway-Based Biomarkers with Crosstalk Analysis for Overall Survival Risk Prediction in Breast Cancer
Source: Front Genet. 2021 Oct 21;12:689715. doi: 10.3389/fgene.2021.689715 (PMC8566719; doi:10.3389/fgene.2021.689715)
Supplement: Supplementary file 15 [file DataSheet1.DOCX]

**Identification of pathway-based biomarkers with crosstalk analysis for overall survival risk prediction in breast cancer**

Xiaohua Liu^1^, Lili Su^2^, Jingcong Li^1^, Guoping Ou^1^*

^1^ State Key Laboratory of Oncology in South China, Sun Yat-sen University Cancer Center, Guangzhou 510060, China

^2^ School of Electronic and Information Engineering, Xi'an Jiaotong University, Xi'an 710049, China

**Correspondence to** Guoping Ou (ouguop@sysucc.org.cn)

**Tel.:** +86 13660065253

**Fax.:** +86 13660065253

**Address:** Dongfeng East Road 651, Guangzhou City, Guangdong Province, 510060, China.

**Competing Interests:** None.

**Abstract**

Recently, many studies have investigated the role of gene-signature on the prognostic assessment of breast cancer (BC), however, the tumor heterogeneity and sequencing noise have limited the clinical usage of these models. Pathway-based approaches are more stable to the perturbation of certain gene expression. In this study, we constructed a prognostic classifier based on survival-related pathway crosstalk analysis. We estimated pathways' deregulation scores (PDSs) for samples collected from public databases to select survival-related pathways. After pathway crosstalk analysis, we conducted K-means clustering analysis to cluster the patients into G1 and G2 subgroups. The survival outcome of the G2 subgroup was significantly worse than the G1 subgroup. Internal and external dataset exhibits high consistency with the training dataset. Significant differences were found between G2 and G1 subgroups on pathway activity, gene mutation, immune cell infiltration levels, and in particular immune cells/pathways' activities were significantly negatively associated with BC patients' outcomes. In conclusion, we established a novel classifier reflecting the overall survival risk of BC and successfully validated its clinical usage on multiple BC datasets, which could offer clinicians inspiration in formulating the clinical treatment plan.

**Keywords:** Breast cancer; pathways' deregulation scores; overall survival risk; classifier

**Introduction**

As a highly metastatic and invasive malignant tumor with high incidence, breast cancer (BC) seriously threatens women's health and quality of life (1-3). BC occupied a quarter of all malignant tumors, which has received numerous clinical attention worldwide (4). At present, the primary treatment options for BC are chemotherapy, surgery, and radiotherapy (5). However, BCs tend to exhibit drug resistance and high recurrence rates on account of heterogeneity, making the therapeutic effects and prognosis of the disease unsatisfactory (6). Screening biomarkers for BC has a significant effect on reducing mortality, early diagnosis, and the improvement of prognosis in BC.

With the development of RNA-Seq high-throughput sequencing technology, various gene expression profiles of BC have been accumulated. Plenty of excellent models have been constructed to decode BC, the majority of them were built based on a single gene list. For example, Vijver et al. (7) established a 70-gene prognosis profile to classify 295 BC patients, which is a powerful predictor for monitoring the prognosis of young BC patients. Tekpli et al. (8) identified clinically relevant immune clusters by integrating 15 BC cohorts, and discovered that patients with pro-tumorigenic immune infiltration were associated with poor prognosis. PAM50 (9) gene signature is a well-known molecular subtyping signature for BC, which could classify the BC into five molecular intrinsic subtypes: Normal-like, Basal-like, HER2+, Luminal A, and Luminal B. These efforts have helped us gain a deeper understanding of BC. Nevertheless, studies have found that due to the tumor heterogeneity and sequencing noise, gene-based signatures were highly unstable and the identified biomarkers were dramatically affected by the selection of training datasets (10, 11). In recent years many researchers indicated pathways could be helpful to extract more stable and interpretable features for risk prediction. Efforts have been made to decode cancer at levels of predefined pathways available in biological databases, such as Kyoto Encyclopedia of Genes and Genomes (KEGG) (12), Reactome (13), and Gene-Set Enrichment Analysis (GSEA) (14). However, most existing pathways are general rather than disease-specific, and disease progression can only be partially affected by them. For pathway pairs with many common genes, we call it crosstalk. Taking the impact of overlapping genes on the pathway activity score (PAS) quantification of the two pathways into consideration can help identify disease-related features. Although it is intuitively believed that pathways will influence each other, especially when genes are shared, the existence of this phenomenon has not been studied in PAS estimation. And few studies have explored the PAS in cancer with crosstalk accommodated among well-established pathways to identify cancer-specific sub-pathways that could be used to predict the prognosis of cancer patients. Therefore, subtyping patients based on PAS and pathway crosstalk analysis is essential to promote personalized medicine.

In this study, we constructed a novel classifier reflecting the overall survival risk of BC based on survival-related pathway crosstalk analysis. We calculated the PAS for each pathway obtained from KEGG and GO resources based on the expression matrix. And then investigated the influence of crosstalk between these selected pathways on different cohorts to select the most critical 100 sub-pathways among all cohorts. we further conducted a K-means clustering analysis to cluster the patients into G1 (moderate) and G2 (aggressive) subgroups. Internal and external dataset exhibits high consistency with the training dataset.

**Materials and Methods**

*Data source*

We collected BC gene expression profiles from TCGA and GEO datasets, and the dataset with less than 20 samples or without overall survival information was excluded from our selection. TCGA mRNA expression data (level 3) and clinical features were downloaded from the UCSC Xena webserver (https://xenabrowser.net/datapages), while GSE16446, GSE42568, GSE7390, GSE20711, GSE1456A, GSE1456B and GSE20685 microarray data and relevant clinical information were downloaded from the GEO database (https://www.ncbi.nlm.nih.gov/gds/). After removing normal and non-survival information samples, we finally obtained 1,090 (TCGA), 107 (GSE16446), 104 (GSE42568), 198 (GSE7390), 88 (GSE20711), 159 (GSE1456A), 159 (GSE1456B) and 327 (GSE20685) BC samples for each dataset.

*Pathway Activity Score (PAS)*

The pathway activity score (PAS) for each dataset was calculated based on the method proposed by Bhandari et al. (15). We downloaded all pathways from the gene ontology (GO) database (<http://geneontology.org/>) and generated a new mRNA expression matrix that contains only genes that exist in it for each pathway. After that, for each gene, based on its expression level, we classified the tumors into two subgroups, the samples in the higher group were scored +1, while the others were scored -1. Finally, we averaged all gene scores in this pathway as the pathway activity score for each tumor sample. A higher PAS indicates a higher pathway activity in the sample, and otherwise, a lower score means lower activity in the sample.

*Selection of survival-related sub-pathways*

Based on PASs and survival information, we calculated the log-rank p-value for each pathway by regression analysis. The pathways were then ranked based on the log-rank p-value. To minimize the false positive rate, we used the common significant pathways of these three large breast cancer cohorts, instead of using any single data set. The combined rank of each pathway was determined by the sure independence screening (SIS) method. We further selected the top n pathways for pathway crosstalk analysis. The threshold n was set to 100, which is much bigger than N/log(N), where N is the sample size of each cohort.

Different pathways often share some of the same genes, which can lead to crosstalk in the prognostic associations of different pathways. Considering the influence of overlapping genes on the PAS quantification of the two pathways can help identify cancer-related features. We further identified the crosstalk among the 100 selected survival-related pathways to define sub-pathways related to survival. The crosstalk between two pathways with at least three genes in common could be classified into three types. The overlapped genes between pathway A and pathway B could be defined as P_A_∩P_B_, while the unique genes that specifically exist in pathway A or pathway B were defined as P_A_–(P_A_∩P_B_) and P_B_–(P_A_∩P_B_). Based on this classifier, each pathway pair could generate three sub-pathways.

To make sure each sub-pathway contains enough genes for further analysis, we obtained sub-pathways that consist of at least three genes. The Cox-PH model was used to calculate the survival risk *p*-value based on the recalculated PAS for each sub-pathway. After Bonferroni correction, we identified critical survival-related pathways and sub-pathways (FDR *p*-value < 0.01) for each dataset, and the overlapped pathways were finally adopted for further modeling.

*Model Construction and Evaluation*

With the pathways generated above, we constructed a pathway activity matrix with the row names are sub-pathways and the column names are sample IDs for each dataset. We performed consensus clustering with the pathway features acquired above to classify the TCGA samples and obtained the best cluster number as 2 based on three metrics, including C-index, Brier score, and log-rank p-value to redefine the samples as G1 and G2 subgroups. To predict these two subgroups for other datasets, we used several machine learning methods, including SVM, Adaboost, and Gaussian, to build a prediction model and obtained the best performance based on the pathway activity matrix. The robustness of the model was evaluated in the TCGA testing dataset and several external individual GEO datasets. We further built a classification model using several machine learning methods, including SVM, Adaboost, and Gaussian, based on these labels. We used the grid search to slightly turn the hyperparameters of the classifier. In the cross-validation procedure, TCGA samples were divided into training and testing datasets in a 4:1 ratio, and the training dataset was used to perform 10-fold cross-validation. To predict the GEO dataset, we used all TCGA samples to build the classification model.

We then compared three metrics, including C-index, Brier score, and log-rank p-value, to evaluate the model's performance. These metrics can quantify the proportion of patient pairs in a cohort whose risk prediction is highly consistent with survival outcomes. Usually, a higher C-index indicates more precise prediction performance, and 1 means perfect prediction, while 0.5 means the prediction performance is similar to random prediction. To calculate the C-index, we built a Cox-PH model based on the clustering labels and the patients' survival information in the TCGA training dataset and predicted the survival rate in the testing dataset, which was calculated by the R "survcomp" package. Brier score reflects the mean difference between observed and predicted survival after a certain period in survival analysis, and a lower score means good performance. Log-rank p-value was calculated by the R "survival" package to show the survival difference between the two groups, and a lower score means a more significant survival difference.

*Survival Analysis*

The log-rank test compares the survival difference of two groups at each observed event time was performed by R “survival” package. Kaplan-Meier analysis was applied to obtain a survival-curve plot of BRCA subtypes. Multivariate Cox regression analysis determined the independent role of this newly established predictor. Besides, we adopted Fisher's exact test to compare the census gene mutation differences between G2 and G1 subgroups in the TCGA cohort. We also compared the distributions of G2 and G1 in different clinicopathological features, such as tumor stage, new tumor event, and sex, by using Fisher exact tests.

We used the "DESeq2" R package (16) to real the differential expressed genes between G2 and G1 subgroup; the significant DEGs were identified as |LogFoldChange| > 1 and false discovery rate (FDR) < 0.05.

*Gene Set Enrichment Analysis (GSEA)*

GSEA analysis was used to compare the pathway activity difference between G2 and G1, in which the R "ClusterProfier" package was performed. We adopted Kyoto Encyclopedia of Genes and Genomes (KEGG) pathways to perform GSEA analysis and the top 20 significant pathways were displayed.

*Mutation Analysis*

The R ‘maftools’ package was utilized to analyze and visualize the mutation data. The mutation data were compared between one group and the other groups using the chi-square test. A p-value of less than 0.05 was considered significant.

**Results**

*Identification of overall survival risk subtypes in BC*

We obtained seven BC datasets (TCGA, GSE1456A, GSE1456B, GSE7390, GSE16446, GSE20685, GSE20711, and GSE42568) gene expression profiles and available clinical survival information from the TCGA and GEO databases. After calculating the PAS for each pathway obtained from KEGG and GO resources and selecting the survival-related pathways, we investigated the influence of crosstalk between these selected pathways on different cohorts, and then the most critical 100 sub-pathways among all cohorts were identified (**Table S1**). K-means clustering analysis was used to divide the TCGA patients into two subgroups, defined as group 1 (G1, moderate) and group 2 (G2, aggressive) (**Fig. S1A**, and **Table S2**). Notably, patients from the G2 subgroup show significantly worse clinical outcomes (overall survival and relapse-free survival) compared to the G1 subgroup (**Fig. S1B**-**C**; *P* = 0.0053 and *P* = 0.0031, respectively; log-rank test). We further built a classifier based on the TCGA training dataset with the label defined by k-means clustering analysis (**Materials and Methods**).

*Evaluation of the performance*

To evaluate the robustness of OS risk prediction of the model, we tested the model performance on the TCGA testing dataset and several external GEO datasets, including GSE1456A, GSE7390, GSE16446, GSE20685, GSE20711, and GSE42568. As shown in **Fig. 1** and **Table. 1**, the model was stable and exhibited excellent classification capability, indicated by C-index and log-rank *p*-values between G2 and G1. The TCGA test cohort generated a high C-index (0.661), low Brier score (0.179), and significant average log-rank p-value (*p* = 0.00123) on survival difference. In different datasets, our classifier can significantly divide the samples into a good prognostic group and a poor prognostic group, which suggested that the newly developed classifier is able to universally predict the overall survival outcomes for patients with BC.

In order to compare the risk prediction capabilities of our predictor with some other clinical information, we performed univariate cox regression analysis for each clinical information (including age, tumor stage, and PAM50 subtyping) in the TCGA dataset as well as the external validation datasets. As shown in **Fig. 2**, our classifier has a more general prognostic ability than other clinical information (*P* < 0.05 in all datasets). We further introduced several published transcriptomic-based predictors as previous study(17), including the proliferation index (18), interferon-γ (IFNγ) signature score (19) as well as cytolytic activity score (20), and performed a multivariate Cox regression analysis with age, tumor stage, and our classifier (**Fig. S2**). In this analysis, the proliferation index and the IFNγ signature score were estimated as ssGSEA score (21) of each gene signature, respectively, and the cytolytic activity score was calculated as the mean expression level of *GZMA* and *PRF1*(20). Reassuringly, our classifier outperformed these predictors, although the p-value of our classifier did not reach statistical significance. In addition, we also test the risk prediction performance in different subgroups of age and tumor stage (**Fig. S3**). This result suggests that our classifier can be used essentially for the typing of different clinical subgroups, although in the low-age group and low-level group of TCGA the p-values did not reach significance.

*Association between different survival subtypes and genomic feature*

We found that the mutation rates of *PI3KCA* and *CDH1* were significantly higher in the G1 group than G2 group (*PI3KCA*: OR = 0.655, 95%CI: 0.471-0.907, *p* = 0.00951; *CDH1*: OR = 0.389, 95%CI: 0.220-0.660, *p* = 0.000195, **Fig. 3**, and **Table S3**, Fisher’s exact test). Other significant differentially mutated genes between the two groups, including *ATR*, *ALK*, *TBX3*, *AKAP9*, *TPR*, *KDM6A*, *CREBBP*, *AMER1*, *CRNKL1*, *TRIM24*, *ZNF429*, *AFF3*, *IGF2BP2*, and *LIFR* (**Table S3**, all p < 0.05). No significant tumor mutation burden (TMB) level difference was found between G1 and G2 subgroups (**Fig. S4**). *PI3KCA* and *CDH1* are two frequently mutated genes in many cancers, including breast cancer, gastric cancer, colorectal carcinoma, and head and neck squamous cell carcinoma (22-25). However, the association of PIK3CA mutation and prognosis has not been clarified clearly, PI3KCA mutation can be associated with a better prognosis (26-29) or a worse prognosis (30, 31). In some studies, PIK3CA mutation even has no obvious relationship with the prognosis (32-37). A similar phenomenon was found for CDH1 mutation as well (38).

We then performed differential expression analysis between G2 and G1 subgroups, and identified 290 upregulated and 824 downregulated genes (|log_2_fold change| > 1 and FDR *p*-value > 0.05) (**Fig. 4A-B**, and **Table S4**) based on the TCGA cohort. KEGG pathway analysis indicated that these upregulated genes were mostly enriched in neuroactive ligand−receptor interaction, cholinergic synapse and estrogen signaling pathways (**Fig. 4C**). The downregulated genes were mostly enriched in immune-related pathways, such as cytokine−cytokine receptor interaction, hematopoietic cell lineage, graft−versus−host disease, and Th17 cell differentiation (**Fig. 4D**). These results prompted that the G1 subgroup might be immune activated subtype, which could be associated with its better overall survival. We also tested the correlations between the two survival subtypes (G2 and G1) and clinicopathological characteristics from the TCGA cohort and found that no significant differences were revealed in age, sex, tumor stage, metastasis coded, estrogen receptor status, progesterone receptor status, and histological type subgroups, instead of PAM50 subtype (**Fig. S5, Table S5**)

We then performed GSEA analysis to compare the G2 and G1 subgroups, aiming to identify critical pathways that displayed different activities between the G1 and G2 subgroups(**Fig. S6, Table S6-8**). Hallmark pathway enrichment analysis showed that immune-related pathways including the inflammatory response, allograft rejection, interferon-gamma response and TNFA-signaling *via* NFκB were enriched in the G1 subgroup, while metabolic-related pathways such as oxidative phosphorylation signaling were activated in the G2 subgroup (**Table S6**). Pathway enrichment analysis indicated that the differences between these two groups were concentrated in the KEGG pathways of “Graft *vs.* host disease”, “primary immunodeficiency”, and “allograft rejection” (**Table S7**) and Reactome pathways related to co-stimulation by the CD28 family, generation of second messenger molecules, and cytokine signaling in the immune system (**Table S8**). Previous studies have proved that metabolic pathway activities like oxidative phosphorylation signaling were negatively correlated with immune infiltration and contributed to a worse prognosis in TNBC (39), which is consistent with our results.

*Comparison of tumor microenvironment between G2 and G1*

We further employed the CIBERSORT algorithm to investigate the distributions of infiltrated immune cells between the G2 and G1 subgroups (**Fig. S7)**. The result revealed that significant differences were obtained between two groups in CD8+T cells, CD4+T memory cells (resting), CD4+T memory (activated), γδ T cells, Macrophages M0, Macrophages M1, Macrophages M2, Dendritic cells (resting), and Mast cells (resting) (**Fig. 5A**). Among macrophages, Macrophages M1 accounts for a higher proportion of the G1 subgroup, while the G2 subgroup consists of a higher proportion of Macrophages M2. Macrophages M2 was found to be dominant in BC and associated with poor clinical outcomes of BC (40), which could be the reason that the G2 subgroup patients have a worse overall survival.

Considering that the tumor tissue has tumor cells, stromal cells and immune cells, we measured stromal score and immune score based on specific gene expression signature to represent the level of immune infiltration and stroma infiltration of each tumor following the previous reported method --- ESTIMATE (41). Also, an ESTIMATE score also calculated which reflects the overall level of both immune infiltration and stromal infiltration. As shown in **Fig. 5B-D**, G1 presented a higher stromal score, immune score and ESTIMATE score compared with G2. These results consistent with the previous definition that the G1 subgroup might be immune activated subtype, there was abundant crosstalk in the tumor microenvironment of this type of tumor, which could benefit from immunotherapy.

**Discussion**

In the era of personalized medicine, there is an urgent need for a molecular marker-based approach to predict the prognostic outcomes of cancer patients accurately. Previous studies have reported many gene-based signatures to subtype BC (7, 42) (8) (9). Here, we constructed an overall survival risk model to classify samples into two subgroups. Internal and external datasets validation exhibits high consistency with the training dataset. Significant differences were found between the G2 and G1 subgroups including pathway activity, gene mutation, immune cell infiltration levels. In particular, immune cells/pathways' activities were significantly negatively associated with BC patients' outcomes.

In order to test whether our classifier is applicable to all ages and tumor grades, we performed prognostic association analysis for different clinical subgroups. For a data set with sufficient samples (more than 20 samples for each subgroup), our classifier can basically distinguish patients with different overall survival periods, although the high-age group and the low-stage group of TCGA have not reached statistical significance. Although the p-value of the high-age group of TCGA does not reach statistical significance (0.076), a clear trend can still be seen. However, it is challenging to explain why our classifier is unable to distinguish OS in the low-stage samples of TCGA with prognostic significance, though it performed well in the other two verification sets (**Fig. S3M and O**).

We found a significant mutation rate difference of PI3KCA and CDH1 gene between the G1 and G2 subgroups. It is not yet clear whether PIK3CA mutation is associated with clinical outcome, PI3KCA mutation can be associated with a better prognosis (26-29) or a worse prognosis (30, 31). In some studies, PIK3CA mutation even has no obvious relationship with the prognosis (32-37). A similar phenomenon was found for CDH1 mutation as well (38). Our results suggested that mutations of PI3KCA are positively associated with a favorable prognosis, but future studies are needed to investigate the potential mechanisms.

We also found that the G1 subgroup displayed significant higher level of immune infiltration, stromal infiltration level than the G2 subgroup. As reported, Th1 and cytotoxic types of memory T cells and CD8+ T cells can predict better prognosis in diverse cancers(43-45). Several studies showed that the existence of mature antigen-presenting dendritic cells (DCs) could infiltrate colon cancer and theoretically increase immune response, which are correlated with improved survival as well (46). Other immune cells such as macrophages always produce plenty of factors influencing tumor cells' survival and growth, chemotaxis, cell invasion, angiogenesis, or repress T cell responses (47). Therefore, a high rate of tumor-associated macrophages typically serves as a poor prognostic factor. It is valuable to predict the efficacy of specific therapies, especially immunotherapy. For example, Peng et al. recently developed a computational method named TIDE to accurately predict immunotherapy outcomes of melanoma(48). The level of immune infiltration was significantly associated with the efficacy of immunotherapy(49). The significant immunological differences between G1 and G2 suggest that our classifier may be predictive of immunotherapy efficacy. We will collect relevant data resources for more in-depth study in our future work.

**Authors' contribution**

Xiaohua Liu and Guoping Ou designed this experiment. Xiaohua Liu and Lili Su performed the research and analyzed the data. Xiaohua Liu and Jingcong Li wrote the manuscript, and Xiaohua Liu, Lili Su, Jingcong Li, and Guoping Ou revised the manuscript. All these authors read and approved the final manuscript.

**Competing Interest**

None.

**Reference:**

1. U. Veronesi, P. Boyle, A. Goldhirsch, R. Orecchia and G. Viale: Breast cancer. *Lancet*, 365(9472), 1727-41 (2005) doi:10.1016/S0140-6736(05)66546-4

2. J. Ruschoff, A. Lebeau, P. Sinn, H. U. Schildhaus, T. Decker, J. Ammann, C. Kunzel, W. Koch and M. Untch: Statistical modelling of HER2-positivity in breast cancer: Final analyses from two large, multicentre, non-interventional studies in Germany. *Breast*, 49, 246-253 (2020) doi:10.1016/j.breast.2019.12.005

3. R. L. Siegel, K. D. Miller and A. Jemal: Cancer statistics, 2019. *CA Cancer J Clin*, 69(1), 7-34 (2019) doi:10.3322/caac.21551

4. J. Ferlay, I. Soerjomataram, R. Dikshit, S. Eser, C. Mathers, M. Rebelo, D. M. Parkin, D. Forman and F. Bray: Cancer incidence and mortality worldwide: sources, methods and major patterns in GLOBOCAN 2012. *Int J Cancer*, 136(5), E359-86 (2015) doi:10.1002/ijc.29210

5. W. Shi, Y. Luo, D. Zhao, H. Huang and W. Pang: Evaluation of the benefit of post-mastectomy radiotherapy in patients with early-stage breast cancer: A propensity score matching study. *Oncol Lett*, 17(6), 4851-4858 (2019) doi:10.3892/ol.2019.10197

6. K. Natarajan, Y. Xie, M. R. Baer and D. D. Ross: Role of breast cancer resistance protein (BCRP/ABCG2) in cancer drug resistance. *Biochem Pharmacol*, 83(8), 1084-103 (2012) doi:10.1016/j.bcp.2012.01.002

7. M. J. van de Vijver, Y. D. He, L. J. van't Veer, H. Dai, A. A. Hart, D. W. Voskuil, G. J. Schreiber, J. L. Peterse, C. Roberts, M. J. Marton, M. Parrish, D. Atsma, A. Witteveen, A. Glas, L. Delahaye, T. van der Velde, H. Bartelink, S. Rodenhuis, E. T. Rutgers, S. H. Friend and R. Bernards: A gene-expression signature as a predictor of survival in breast cancer. *N Engl J Med*, 347(25), 1999-2009 (2002) doi:10.1056/NEJMoa021967

8. X. Tekpli, T. Lien, A. H. Røssevold, D. Nebdal, E. Borgen, H. O. Ohnstad, J. A. Kyte, J. Vallon-Christersson, M. Fongaard, E. U. Due, L. G. Svartdal, M. A. T. Sveli, Ø. Garred, A. Frigessi, K. K. Sahlberg, T. Sørlie, H. G. Russnes, B. Naume and V. N. Kristensen: An independent poor-prognosis subtype of breast cancer defined by a distinct tumor immune microenvironment. *Nat Commun*, 10(1), 5499 (2019) doi:10.1038/s41467-019-13329-5

9. J. S. Parker, M. Mullins, M. C. Cheang, S. Leung, D. Voduc, T. Vickery, S. Davies, C. Fauron, X. He, Z. Hu, J. F. Quackenbush, I. J. Stijleman, J. Palazzo, J. S. Marron, A. B. Nobel, E. Mardis, T. O. Nielsen, M. J. Ellis, C. M. Perou and P. S. Bernard: Supervised risk predictor of breast cancer based on intrinsic subtypes. *J Clin Oncol*, 27(8), 1160-7 (2009) doi:10.1200/jco.2008.18.1370

10. S. Michiels, S. Koscielny and C. Hill: Prediction of cancer outcome with microarrays: a multiple random validation strategy. *Lancet*, 365(9458), 488-92 (2005) doi:10.1016/s0140-6736(05)17866-0

11. E. Domany: Using high-throughput transcriptomic data for prognosis: a critical overview and perspectives. *Cancer Res*, 74(17), 4612-21 (2014) doi:10.1158/0008-5472.Can-13-3338

12. M. Kanehisa, Y. Sato, M. Kawashima, M. Furumichi and M. Tanabe: KEGG as a reference resource for gene and protein annotation. *Nucleic Acids Res*, 44(D1), D457-62 (2016) doi:10.1093/nar/gkv1070

13. A. Fabregat, S. Jupe, L. Matthews, K. Sidiropoulos, M. Gillespie, P. Garapati, R. Haw, B. Jassal, F. Korninger, B. May, M. Milacic, C. D. Roca, K. Rothfels, C. Sevilla, V. Shamovsky, S. Shorser, T. Varusai, G. Viteri, J. Weiser, G. Wu, L. Stein, H. Hermjakob and P. D'Eustachio: The Reactome Pathway Knowledgebase. *Nucleic Acids Res*, 46(D1), D649-d655 (2018) doi:10.1093/nar/gkx1132

14. W. He, L. Chen, K. Yuan, Q. Zhou, L. Peng and Y. Han: Gene set enrichment analysis and meta-analysis to identify six key genes regulating and controlling the prognosis of esophageal squamous cell carcinoma. *J Thorac Dis*, 10(10), 5714-5726 (2018) doi:10.21037/jtd.2018.09.55

15. V. Bhandari, C. Hoey, L. Y. Liu, E. Lalonde, J. Ray, J. Livingstone, R. Lesurf, Y.-J. Shiah, T. Vujcic, X. Huang, S. M. G. Espiritu, L. E. Heisler, F. Yousif, V. Huang, T. N. Yamaguchi, C. Q. Yao, V. Y. Sabelnykova, M. Fraser, M. L. K. Chua, T. van der Kwast, S. K. Liu, P. C. Boutros and R. G. Bristow: Molecular landmarks of tumor hypoxia across cancer types. *Nature Genetics*, 51(2), 308-318 (2019) doi:10.1038/s41588-018-0318-2

16. M. I. Love, W. Huber and S. Anders: Moderated estimation of fold change and dispersion for RNA-seq data with DESeq2. *Genome Biol*, 15(12), 550 (2014) doi:10.1186/s13059-014-0550-8

17. J. S. Lee, N. U. Nair, G. Dinstag, L. Chapman, Y. Chung, K. Wang, S. Sinha, H. Cha, D. Kim, A. V. Schperberg, A. Srinivasan, V. Lazar, E. Rubin, S. Hwang, R. Berger, T. Beker, Z. Ronai, S. Hannenhalli, M. R. Gilbert, R. Kurzrock, S. H. Lee, K. Aldape and E. Ruppin: Synthetic lethality-mediated precision oncology via the tumor transcriptome. *Cell*, 184(9), 2487-2502 e13 (2021) doi:10.1016/j.cell.2021.03.030

18. M. L. Whitfield, L. K. George, G. D. Grant and C. M. Perou: Common markers of proliferation. *Nat Rev Cancer*, 6(2), 99-106 (2006) doi:10.1038/nrc1802

19. M. Ayers, J. Lunceford, M. Nebozhyn, E. Murphy, A. Loboda, D. R. Kaufman, A. Albright, J. D. Cheng, S. P. Kang, V. Shankaran, S. A. Piha-Paul, J. Yearley, T. Y. Seiwert, A. Ribas and T. K. McClanahan: IFN-γ-related mRNA profile predicts clinical response to PD-1 blockade. *J Clin Invest*, 127(8), 2930-2940 (2017) doi:10.1172/jci91190

20. M. S. Rooney, S. A. Shukla, C. J. Wu, G. Getz and N. Hacohen: Molecular and genetic properties of tumors associated with local immune cytolytic activity. *Cell*, 160(1-2), 48-61 (2015) doi:10.1016/j.cell.2014.12.033

21. M. Yi, D. V. Nissley, F. McCormick and R. M. Stephens: ssGSEA score-based Ras dependency indexes derived from gene expression data reveal potential Ras addiction mechanisms with possible clinical implications. *Sci Rep*, 10(1), 10258 (2020) doi:10.1038/s41598-020-66986-8

22. Y. An, J. R. Adams, D. P. Hollern, A. Zhao, S. G. Chang, M. S. Gams, P. E. D. Chung, X. He, R. Jangra, J. S. Shah, J. Yang, L. A. Beck, N. Raghuram, K. J. Kozma, A. J. Loch, W. Wang, C. Fan, S. J. Done, E. Zacksenhaus, C. J. Guidos, C. M. Perou and S. E. Egan: Cdh1 and Pik3ca Mutations Cooperate to Induce Immune-Related Invasive Lobular Carcinoma of the Breast. *Cell Rep*, 25(3), 702-714.e6 (2018) doi:10.1016/j.celrep.2018.09.056

23. S. Hansford, P. Kaurah, H. Li-Chang, M. Woo, J. Senz, H. Pinheiro, K. A. Schrader, D. F. Schaeffer, K. Shumansky, G. Zogopoulos, T. A. Santos, I. Claro, J. Carvalho, C. Nielsen, S. Padilla, A. Lum, A. Talhouk, K. Baker-Lange, S. Richardson, I. Lewis, N. M. Lindor, E. Pennell, A. MacMillan, B. Fernandez, G. Keller, H. Lynch, S. P. Shah, P. Guilford, S. Gallinger, G. Corso, F. Roviello, C. Caldas, C. Oliveira, P. D. Pharoah and D. G. Huntsman: Hereditary Diffuse Gastric Cancer Syndrome: CDH1 Mutations and Beyond. *JAMA Oncol*, 1(1), 23-32 (2015) doi:10.1001/jamaoncol.2014.168

24. S. Z. Millis, S. Ikeda, S. Reddy, Z. Gatalica and R. Kurzrock: Landscape of Phosphatidylinositol-3-Kinase Pathway Alterations Across 19 784 Diverse Solid Tumors. *JAMA Oncol*, 2(12), 1565-1573 (2016) doi:10.1001/jamaoncol.2016.0891

25. Y. Zhang, P. Kwok-Shing Ng, M. Kucherlapati, F. Chen, Y. Liu, Y. H. Tsang, G. de Velasco, K. J. Jeong, R. Akbani, A. Hadjipanayis, A. Pantazi, C. A. Bristow, E. Lee, H. S. Mahadeshwar, J. Tang, J. Zhang, L. Yang, S. Seth, S. Lee, X. Ren, X. Song, H. Sun, J. Seidman, L. J. Luquette, R. Xi, L. Chin, A. Protopopov, T. F. Westbrook, C. S. Shelley, T. K. Choueiri, M. Ittmann, C. Van Waes, J. N. Weinstein, H. Liang, E. P. Henske, A. K. Godwin, P. J. Park, R. Kucherlapati, K. L. Scott, G. B. Mills, D. J. Kwiatkowski and C. J. Creighton: A Pan-Cancer Proteogenomic Atlas of PI3K/AKT/mTOR Pathway Alterations. *Cancer Cell*, 31(6), 820-832.e3 (2017) doi:10.1016/j.ccell.2017.04.013

26. M. Barbareschi, F. Buttitta, L. Felicioni, S. Cotrupi, F. Barassi, M. Del Grammastro, A. Ferro, P. Dalla Palma, E. Galligioni and A. Marchetti: Different prognostic roles of mutations in the helical and kinase domains of the PIK3CA gene in breast carcinomas. *Clin Cancer Res*, 13(20), 6064-9 (2007) doi:10.1158/1078-0432.Ccr-07-0266

27. K. Kalinsky, L. M. Jacks, A. Heguy, S. Patil, M. Drobnjak, U. K. Bhanot, C. V. Hedvat, T. A. Traina, D. Solit, W. Gerald and M. E. Moynahan: PIK3CA mutation associates with improved outcome in breast cancer. *Clin Cancer Res*, 15(16), 5049-59 (2009) doi:10.1158/1078-0432.Ccr-09-0632

28. N. Maruyama, Y. Miyoshi, T. Taguchi, Y. Tamaki, M. Monden and S. Noguchi: Clinicopathologic analysis of breast cancers with PIK3CA mutations in Japanese women. *Clin Cancer Res*, 13(2 Pt 1), 408-14 (2007) doi:10.1158/1078-0432.Ccr-06-0267

29. G. Pérez-Tenorio, L. Alkhori, B. Olsson, M. A. Waltersson, B. Nordenskjöld, L. E. Rutqvist, L. Skoog and O. Stål: PIK3CA mutations and PTEN loss correlate with similar prognostic factors and are not mutually exclusive in breast cancer. *Clin Cancer Res*, 13(12), 3577-84 (2007) doi:10.1158/1078-0432.Ccr-06-1609

30. E. Lerma, L. Catasus, A. Gallardo, G. Peiro, C. Alonso, I. Aranda, A. Barnadas and J. Prat: Exon 20 PIK3CA mutations decreases survival in aggressive (HER-2 positive) breast carcinomas. *Virchows Arch*, 453(2), 133-9 (2008) doi:10.1007/s00428-008-0643-4

31. S. Y. Li, M. Rong, F. Grieu and B. Iacopetta: PIK3CA mutations in breast cancer are associated with poor outcome. *Breast Cancer Res Treat*, 96(1), 91-5 (2006) doi:10.1007/s10549-005-9048-0

32. S. Boyault, Y. Drouet, C. Navarro, T. Bachelot, C. Lasset, I. Treilleux, E. Tabone, A. Puisieux and Q. Wang: Mutational characterization of individual breast tumors: TP53 and PI3K pathway genes are frequently and distinctively mutated in different subtypes. *Breast Cancer Res Treat*, 132(1), 29-39 (2012) doi:10.1007/s10549-011-1518-y

33. Y. L. Lai, B. L. Mau, W. H. Cheng, H. M. Chen, H. H. Chiu and C. Y. Tzen: PIK3CA exon 20 mutation is independently associated with a poor prognosis in breast cancer patients. *Ann Surg Oncol*, 15(4), 1064-9 (2008) doi:10.1245/s10434-007-9751-7

34. S. Loi, B. Haibe-Kains, S. Majjaj, F. Lallemand, V. Durbecq, D. Larsimont, A. M. Gonzalez-Angulo, L. Pusztai, W. F. Symmans, A. Bardelli, P. Ellis, A. N. Tutt, C. E. Gillett, B. T. Hennessy, G. B. Mills, W. A. Phillips, M. J. Piccart, T. P. Speed, G. A. McArthur and C. Sotiriou: PIK3CA mutations associated with gene signature of low mTORC1 signaling and better outcomes in estrogen receptor-positive breast cancer. *Proc Natl Acad Sci U S A*, 107(22), 10208-13 (2010) doi:10.1073/pnas.0907011107

35. A. Michelucci, C. Di Cristofano, A. Lami, P. Collecchi, A. Caligo, N. Decarli, M. Leopizzi, P. Aretini, G. Bertacca, R. P. Porta, S. Ricci, C. Della Rocca, G. Stanta, G. Bevilacqua and A. Cavazzana: PIK3CA in breast carcinoma: a mutational analysis of sporadic and hereditary cases. *Diagn Mol Pathol*, 18(4), 200-5 (2009) doi:10.1097/PDM.0b013e31818e5fa4

36. L. H. Saal, K. Holm, M. Maurer, L. Memeo, T. Su, X. Wang, J. S. Yu, P. O. Malmström, M. Mansukhani, J. Enoksson, H. Hibshoosh, A. Borg and R. Parsons: PIK3CA mutations correlate with hormone receptors, node metastasis, and ERBB2, and are mutually exclusive with PTEN loss in human breast carcinoma. *Cancer Res*, 65(7), 2554-9 (2005) doi:10.1158/0008-5472-can-04-3913

37. K. Stemke-Hale, A. M. Gonzalez-Angulo, A. Lluch, R. M. Neve, W. L. Kuo, M. Davies, M. Carey, Z. Hu, Y. Guan, A. Sahin, W. F. Symmans, L. Pusztai, L. K. Nolden, H. Horlings, K. Berns, M. C. Hung, M. J. van de Vijver, V. Valero, J. W. Gray, R. Bernards, G. B. Mills and B. T. Hennessy: An integrative genomic and proteomic analysis of PIK3CA, PTEN, and AKT mutations in breast cancer. *Cancer Res*, 68(15), 6084-91 (2008) doi:10.1158/0008-5472.Can-07-6854

38. G. Corso, P. Veronesi, V. Sacchini and V. Galimberti: Prognosis and outcome in CDH1-mutant lobular breast cancer. *Eur J Cancer Prev*, 27(3), 237-238 (2018) doi:10.1097/cej.0000000000000405

39. Y. Gong, P. Ji, Y. S. Yang, S. Xie, T. J. Yu, Y. Xiao, M. L. Jin, D. Ma, L. W. Guo, Y. C. Pei, W. J. Chai, D. Q. Li, F. Bai, F. Bertucci, X. Hu, Y. Z. Jiang and Z. M. Shao: Metabolic-Pathway-Based Subtyping of Triple-Negative Breast Cancer Reveals Potential Therapeutic Targets. *Cell Metab*, 33(1), 51-64.e9 (2021) doi:10.1016/j.cmet.2020.10.012

40. X. Bao, R. Shi, T. Zhao, Y. Wang, N. Anastasov, M. Rosemann and W. Fang: Integrated analysis of single-cell RNA-seq and bulk RNA-seq unravels tumour heterogeneity plus M2-like tumour-associated macrophage infiltration and aggressiveness in TNBC. *Cancer Immunol Immunother*, 70(1), 189-202 (2021) doi:10.1007/s00262-020-02669-7

41. K. Yoshihara, M. Shahmoradgoli, E. Martinez, R. Vegesna, H. Kim, W. Torres-Garcia, V. Trevino, H. Shen, P. W. Laird, D. A. Levine, S. L. Carter, G. Getz, K. Stemke-Hale, G. B. Mills and R. G. Verhaak: Inferring tumour purity and stromal and immune cell admixture from expression data. *Nat Commun*, 4, 2612 (2013) doi:10.1038/ncomms3612

42. M. Pu, K. Messer, S. R. Davies, T. L. Vickery, E. Pittman, B. A. Parker, M. J. Ellis, S. W. Flatt, C. R. Marinac, S. H. Nelson, E. R. Mardis, J. P. Pierce and L. Natarajan: Research-based PAM50 signature and long-term breast cancer survival. *Breast Cancer Res Treat*, 179(1), 197-206 (2020) doi:10.1007/s10549-019-05446-y

43. M. St. Paul and P. S. Ohashi: The Roles of CD8+ T Cell Subsets in Antitumor Immunity. *Trends in Cell Biology*, 30(9), 695-704 (2020) doi:<https://doi.org/10.1016/j.tcb.2020.06.003>

44. M. Wei, D. Shen, S. Mulmi Shrestha, J. Liu, J. Zhang and Y. Yin: The Progress of T Cell Immunity Related to Prognosis in Gastric Cancer. *BioMed Research International*, 2018, 3201940 (2018) doi:10.1155/2018/3201940

45. S. Yi, Y. Zhang, W. Xiong, W. Chen, Z. Hou, Y. Yang, Y. Yan, Y. Wei, R. Cui, H. Wang, Z. Yu, H. Li, Z. Li, W. Liu, R. Lv, T. Wang, K. Ru, D. Zou, M. Shu, L. Qiu and D. Yu: Prominent immune signatures of T cells are specifically associated with indolent B-cell lymphoproliferative disorders and predict prognosis. *Clinical & Translational Immunology*, 9(1), e01105 (2020) doi:<https://doi.org/10.1002/cti2.1105>

46. T. Schwaab, J. E. Weiss, A. R. Schned and R. J. Barth, Jr.: Dendritic cell infiltration in colon cancer. *J Immunother*, 24(2), 130-7 (2001)

47. F. Pagès, J. Galon, M. C. Dieu-Nosjean, E. Tartour, C. Sautès-Fridman and W. H. Fridman: Immune infiltration in human tumors: a prognostic factor that should not be ignored. *Oncogene*, 29(8), 1093-102 (2010) doi:10.1038/onc.2009.416

48. P. Jiang, S. Gu, D. Pan, J. Fu, A. Sahu, X. Hu, Z. Li, N. Traugh, X. Bu, B. Li, J. Liu, G. J. Freeman, M. A. Brown, K. W. Wucherpfennig and X. S. Liu: Signatures of T cell dysfunction and exclusion predict cancer immunotherapy response. *Nat Med*, 24(10), 1550-1558 (2018) doi:10.1038/s41591-018-0136-1

49. J. Galon and D. Bruni: Approaches to treat immune hot, altered and cold tumours with combination immunotherapies. *Nat Rev Drug Discov*, 18(3), 197-218 (2019) doi:10.1038/s41573-018-0007-y

**Figure and table legend:**

**Fig. 1|** Kaplan-Meier analysis to determine the survival differences between group 2 (G2) and group 1 (G1).

**Fig. 2|** Univariate Cox analysis of the classifier as well as regular clinical classification (Age, PAM50 and tumor stage) in TCGA and other extermal validation cohorts.

**Fig. 3|** Census mutation landscape between group 2 (G2) and group 1 (G1). Only cancer census genes in the COSMIC database are shown in the plot. The significance of the difference in gene mutation frequency between the two groups is shown in the barplot on the right (fisher’s exact test).

**Fig. 4|** Analysis of differentially expressed genes and their corresponding pathways. A. Gene expression differences between group 2 (G2) and group 1 (G1). B. PCA analysis shows the clustering results of group 2 (G2) and group 1 (G1). C and D. KEGG pathway enrichment analysis for the up- (up panel) and down-regulated (down panel) genes between G2 and G1 subgroups.

**Fig. 5|** Tumor microenvironment differences between group 2 (G2) and group 1 (G1) subgroups. A. Comparison of each immune cell component between G1 and G2. B. Comparison of stroma score between G1 and G2. C. Comparison of immune score between G1 and G2. D. Comparison of ESTIMATE score between G1 and G2.

**Fig. S1|** The establishment and preliminary validation of the classifier. A. Heatmap shows the clustering of two subclassification groups. B. Kaplan-Meier plot shows the OS difference between group 2 (G2) and group 1(G1). C. Kaplan-Meier plot shows the RFS difference between group 2 (G2) and group 1(G1).

**Fig. S2|** Multivariate Cox regression analysis of prognostic factors for overall survival in TCGA patients. Age, tumor stage, proliferation score, IFNg score, cytolytic activity as well as our classifier were taken into consideration in this analysis.

**Fig. S3|** KM curves show the prognostic performance of the classifier in different age level and tumor stage level of all included cohorts. Only cohorts with available clinical information are shown in the plot.

**Fig. S4|** Comparison of tumor mutation burden between the G1 and G2 subgroups.

**Fig. S5|** Sankey plot shows the distribution of group 2 (G2) and group 1 (G1) in different clinicopathological and genetic subgroups.

**Fig. S6|** Gene Set Enrichment Analysis (GSEA) analysis shows the differences between group 2 (G2) and group 1 (G1) at Hallmark, (Kyoto Encyclopedia of Genes and Genomes) KEGG, and Reactome pathway aspects.

**Fig. S7|** CIBERSORT analysis shows the distributions of 22 tumor infiltrated immune cells in each breast cancer sample.

**Table S1|** Pathway identifiers and their corresponding genes used in the current study.

**Table S2|** Performance of the classifier in predicting overall survival of breast cancer patients in different cohorts.

**Table S3|** Significantly mutated census genes between group 2 (G2) and group 1 (G1) subgroups.

**Table S4|** Differentially expressed genes between group 2 (G2) and group 1 (G1) subgroups.

**Table S5|** Statistical analysis for the distributions of group 2 (G2) and group 1 (G1) subgroups in different clinicopathological and genetic subgroups.

**Table S6|** Detailed information of hallmark Gene Set Enrichment Analysis (GSEA) analysis.

**Table S7|** Detailed information of (Kyoto Encyclopedia of Genes and Genomes) KEGG Gene Set Enrichment Analysis (GSEA) analysis.

**Table S8|** Detailed information of Reactome Gene Set Enrichment Analysis (GSEA) analysis.

**Table 1|** Cross-validation based performance robustness of classifier on TCGA training and testing cohorts.

| **Cohorts** | **Omics Type** | **Samples** | **C-index** | **Brier Score** | **Log-rank *p*** |
| --- | --- | --- | --- | --- | --- |
| TCGA_test | RNA-Seq | 218 | 0.661 | 0.179 | 1.23E-03 |
| GSE1456A | Microarray | 159 | 0.600 | 0.122 | 3.35E-03 |
| GSE1456B | Microarray | 159 | 0.599 | 0.121 | 6.78E-03 |
| GSE16446 | Microarray | 107 | 0.646 | 0.112 | 4.30E-03 |
| GSE20685 | Microarray | 327 | 0.579 | 0.153 | 3.96E-03 |
| GSE20711 | Microarray | 88 | 0.656 | 0.189 | 1.49E-03 |
| GSE42568 | Microarray | 104 | 0.679 | 0.195 | 6.27E-05 |
| GSE7390 | Microarray | 198 | 0.597 | 0.180 | 1.03E-02 |

TCGA, The Cancer Genome Atlas
